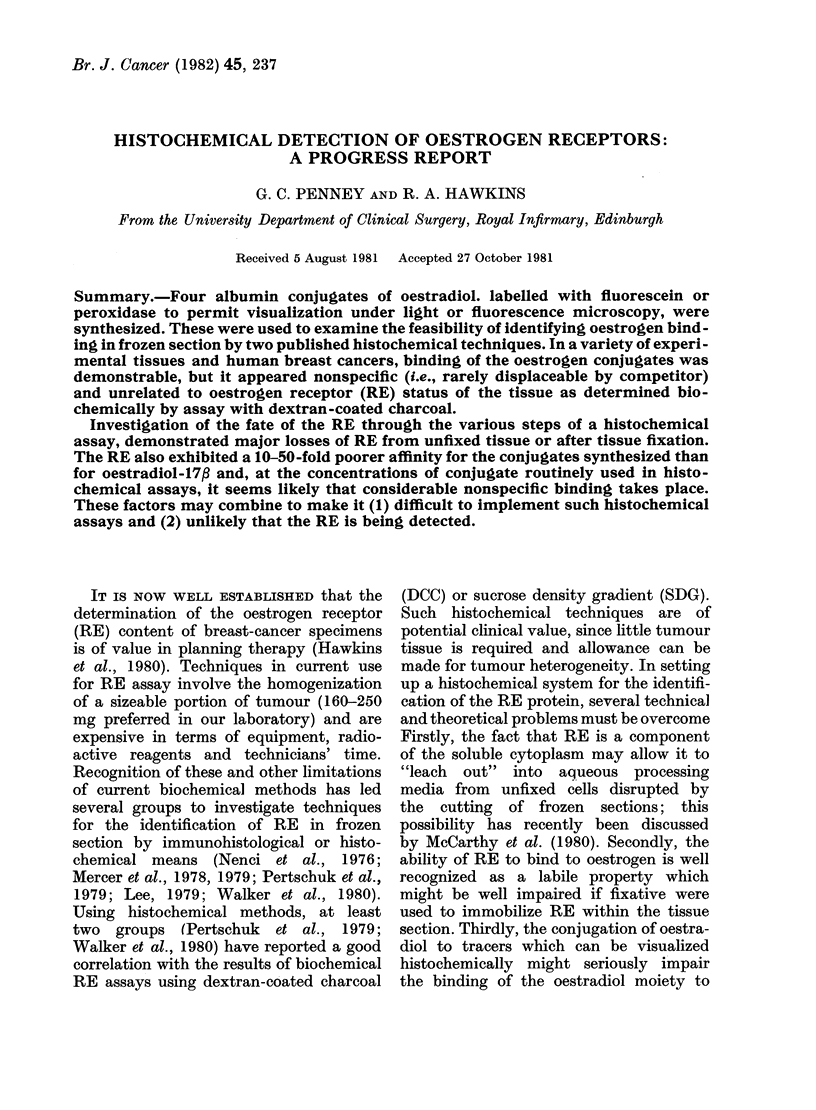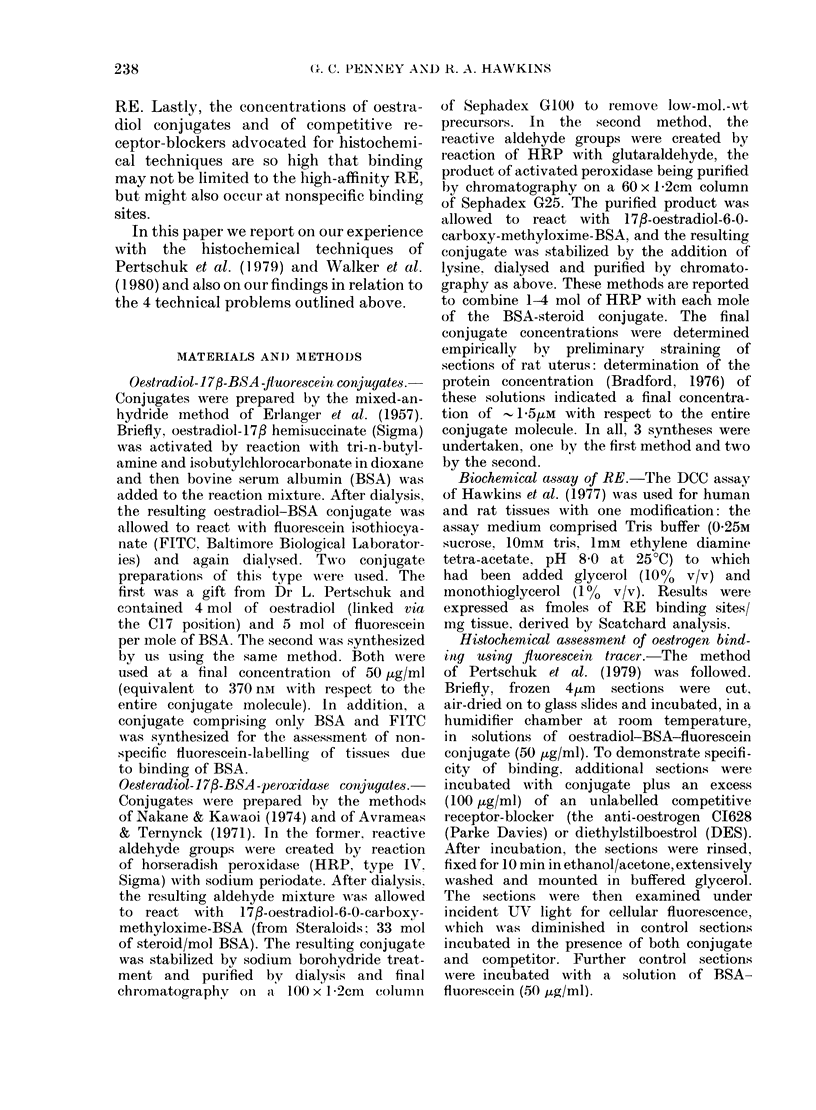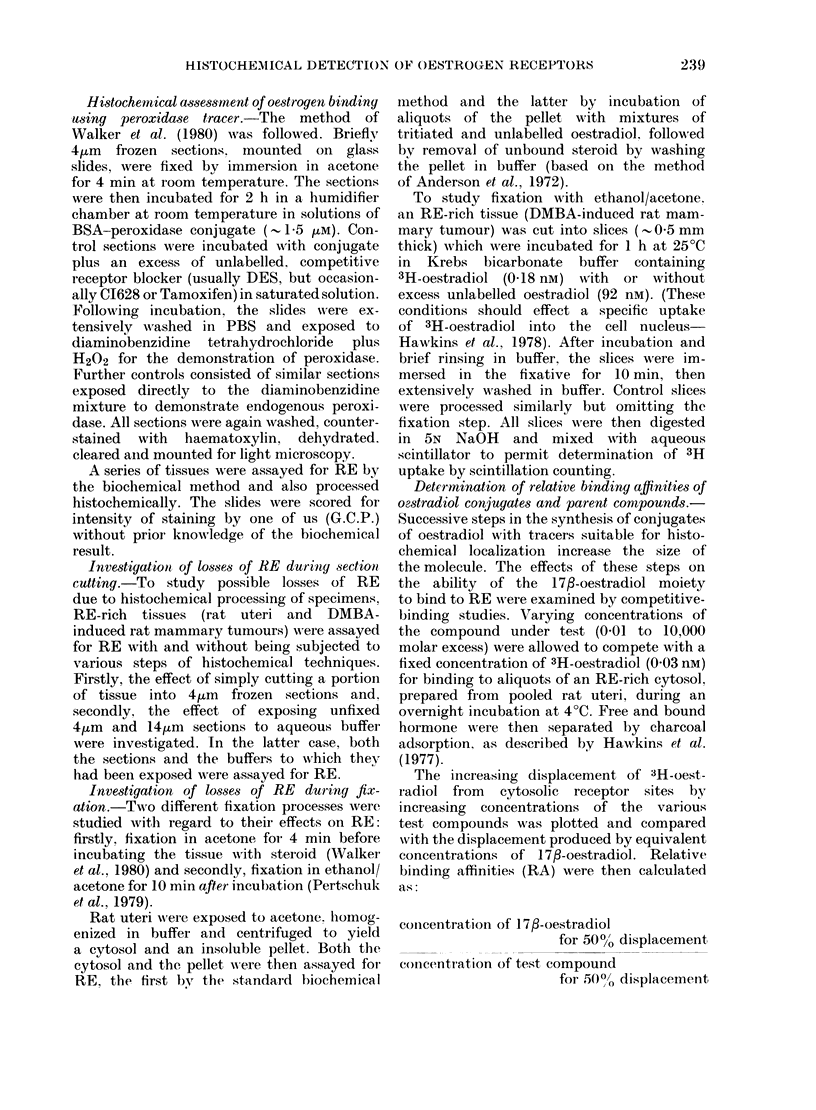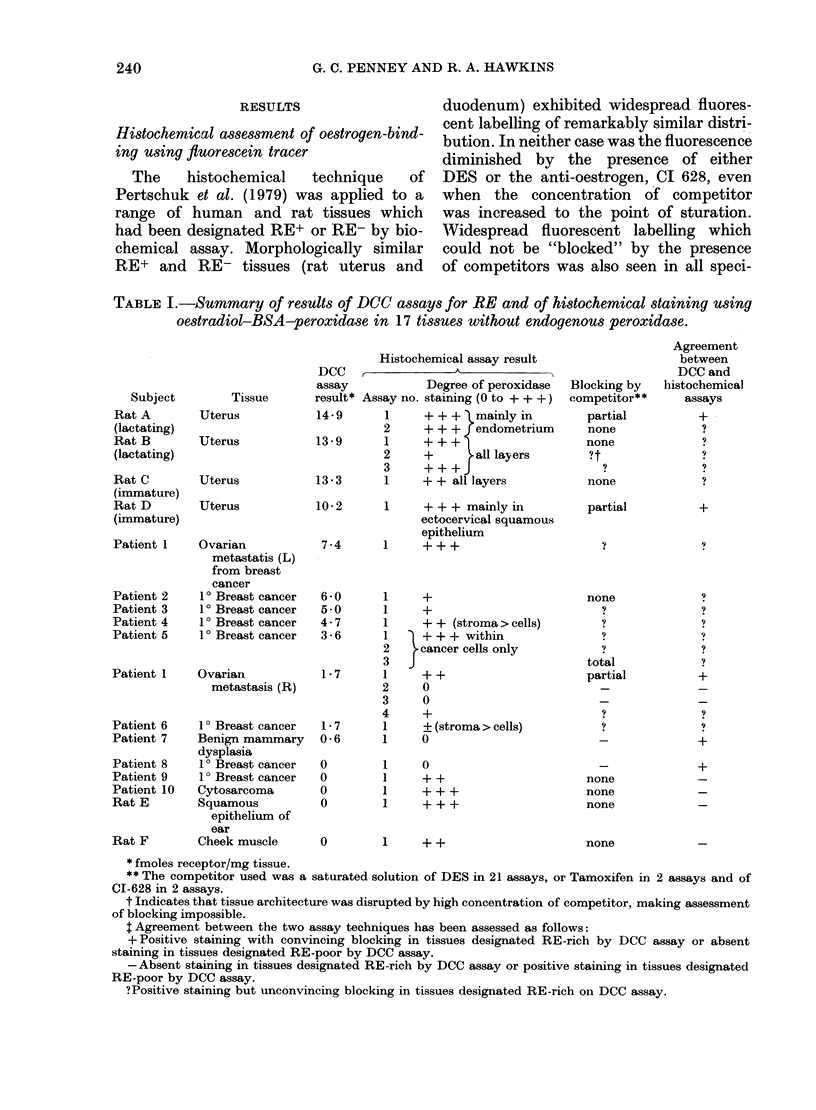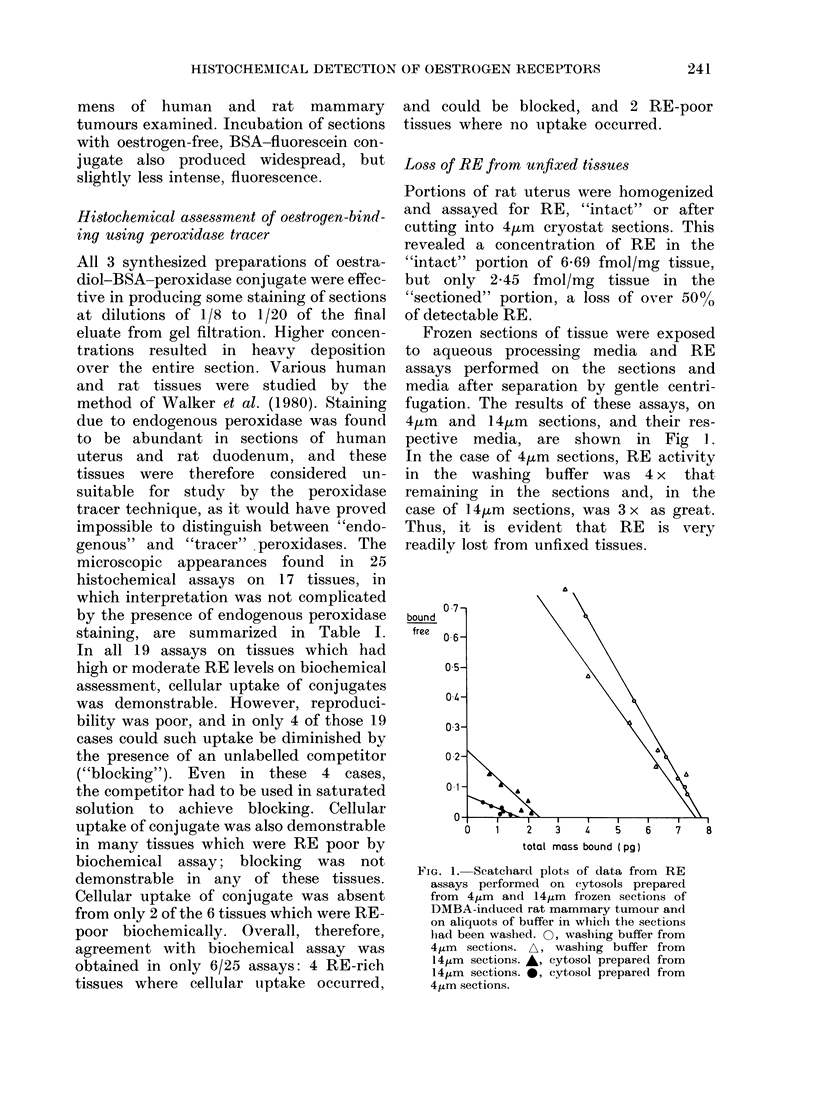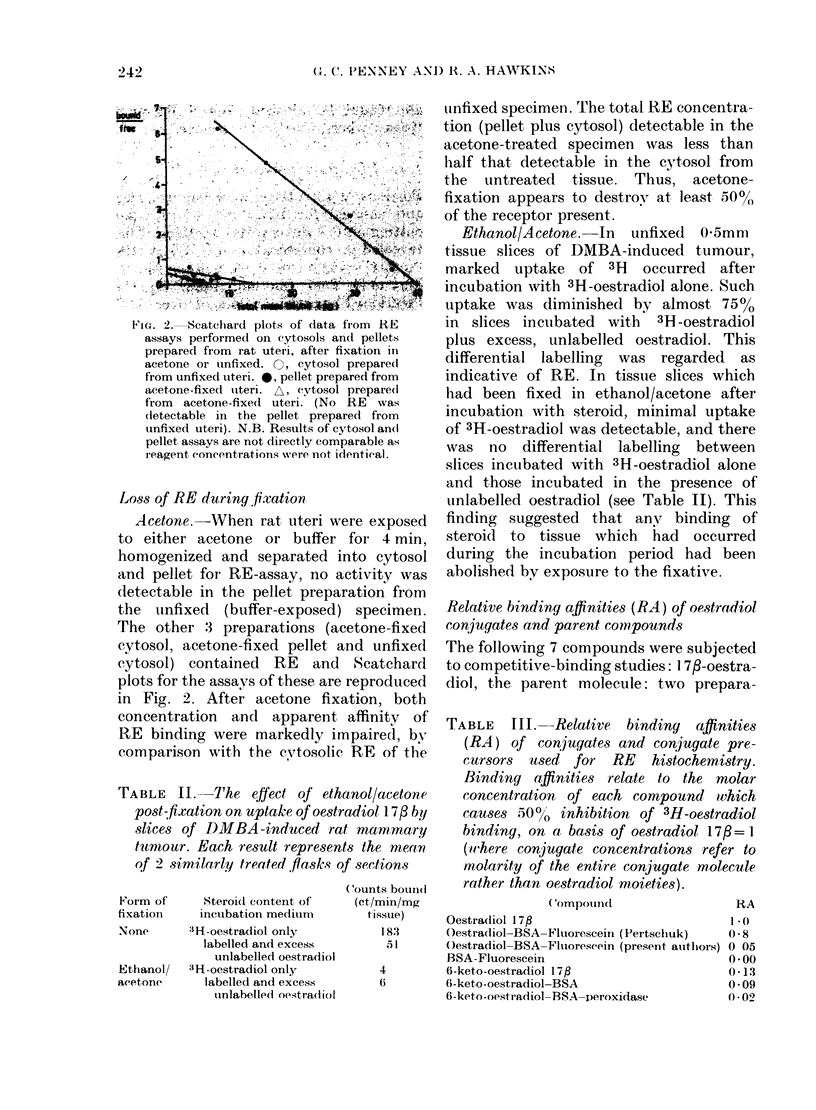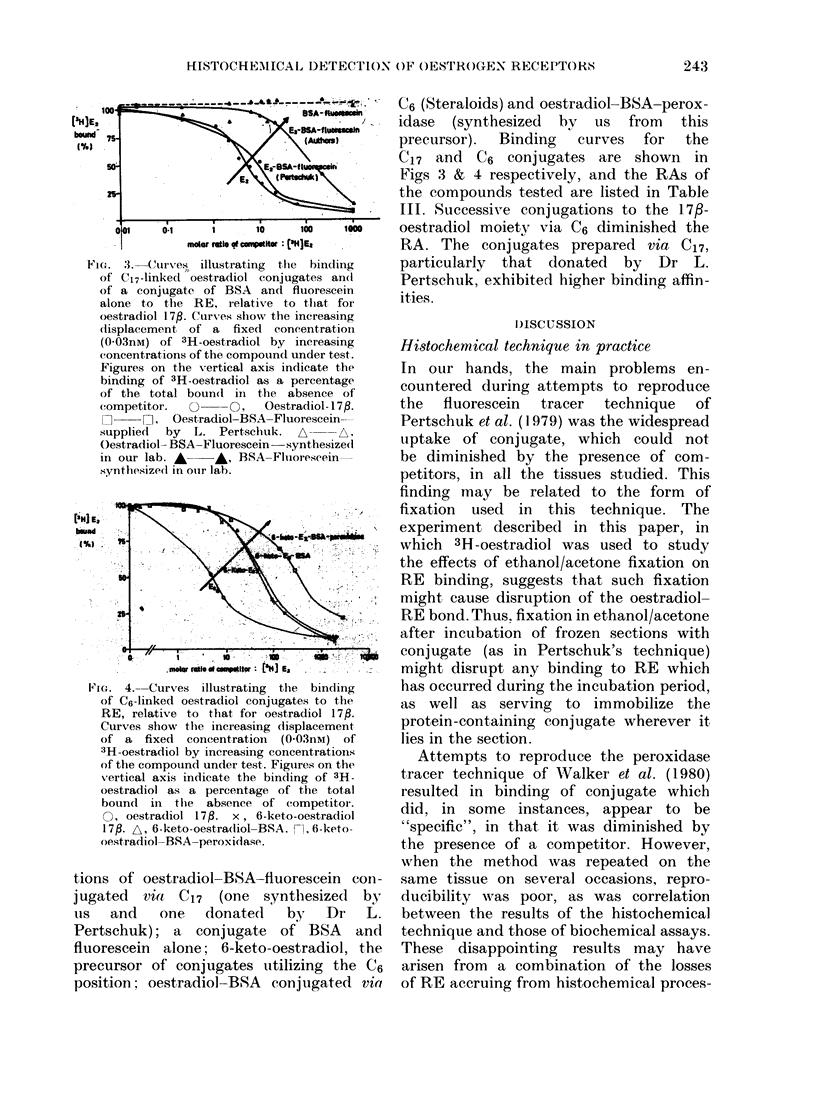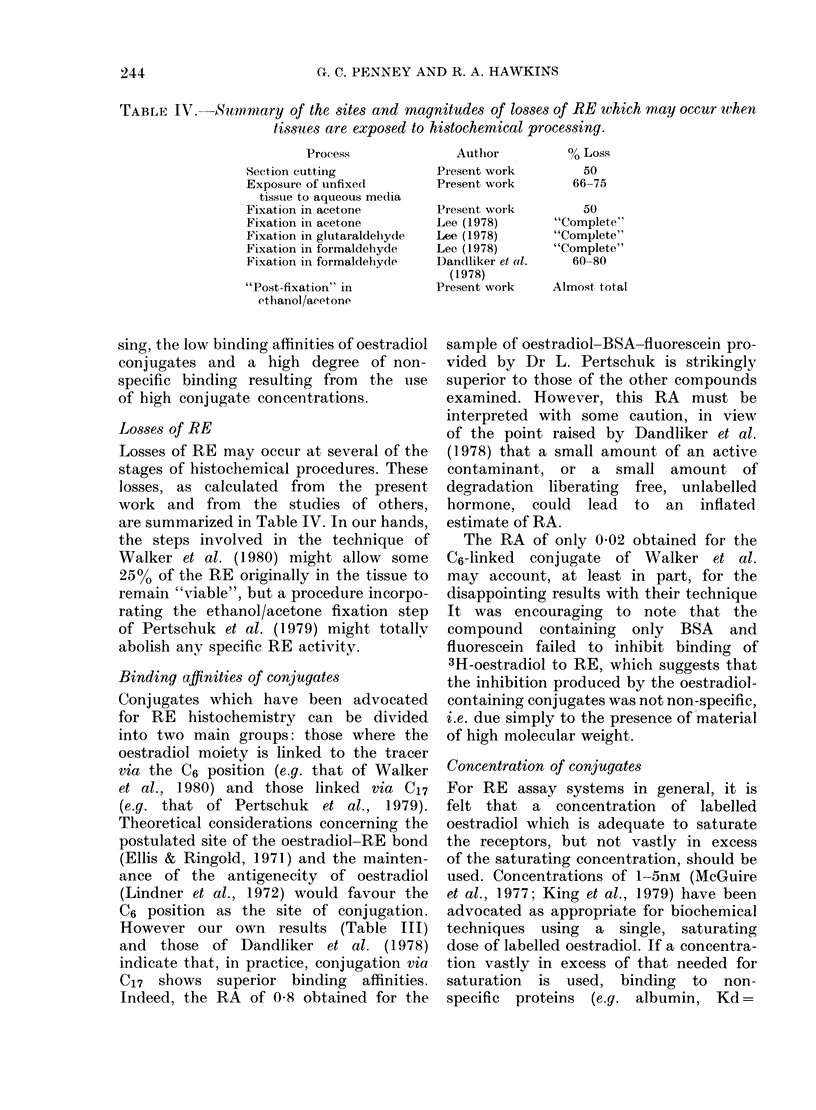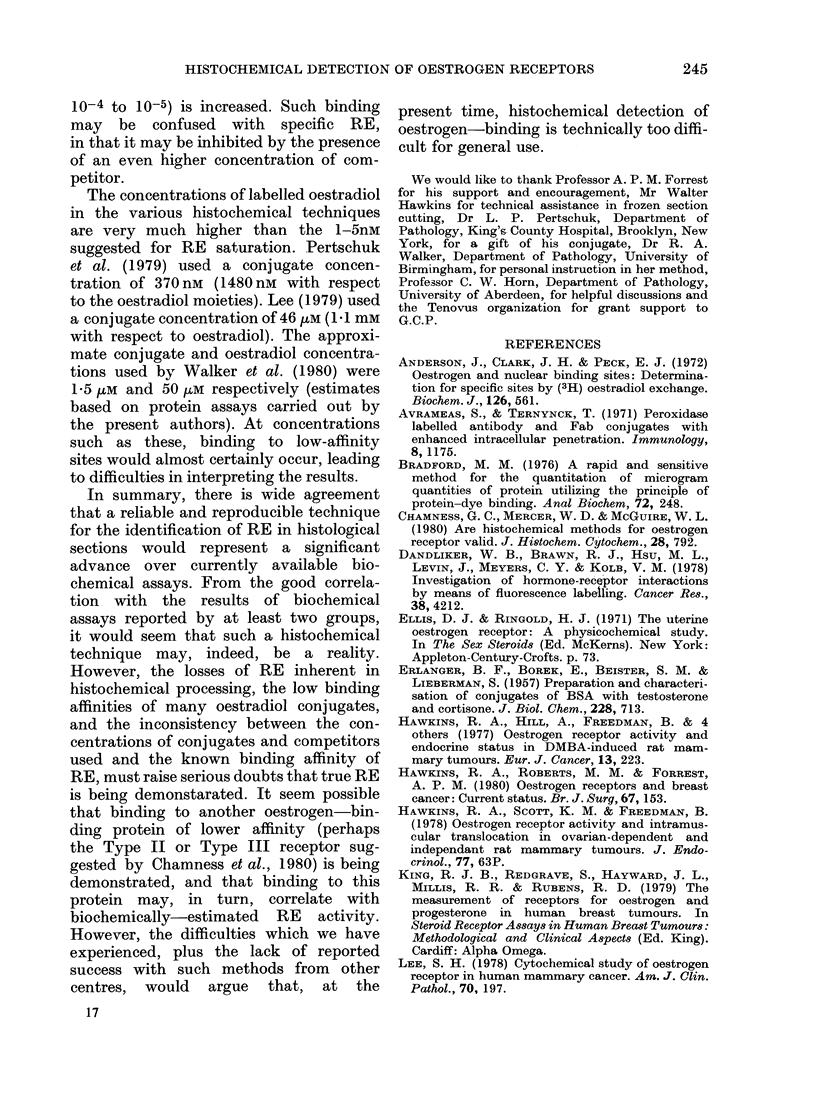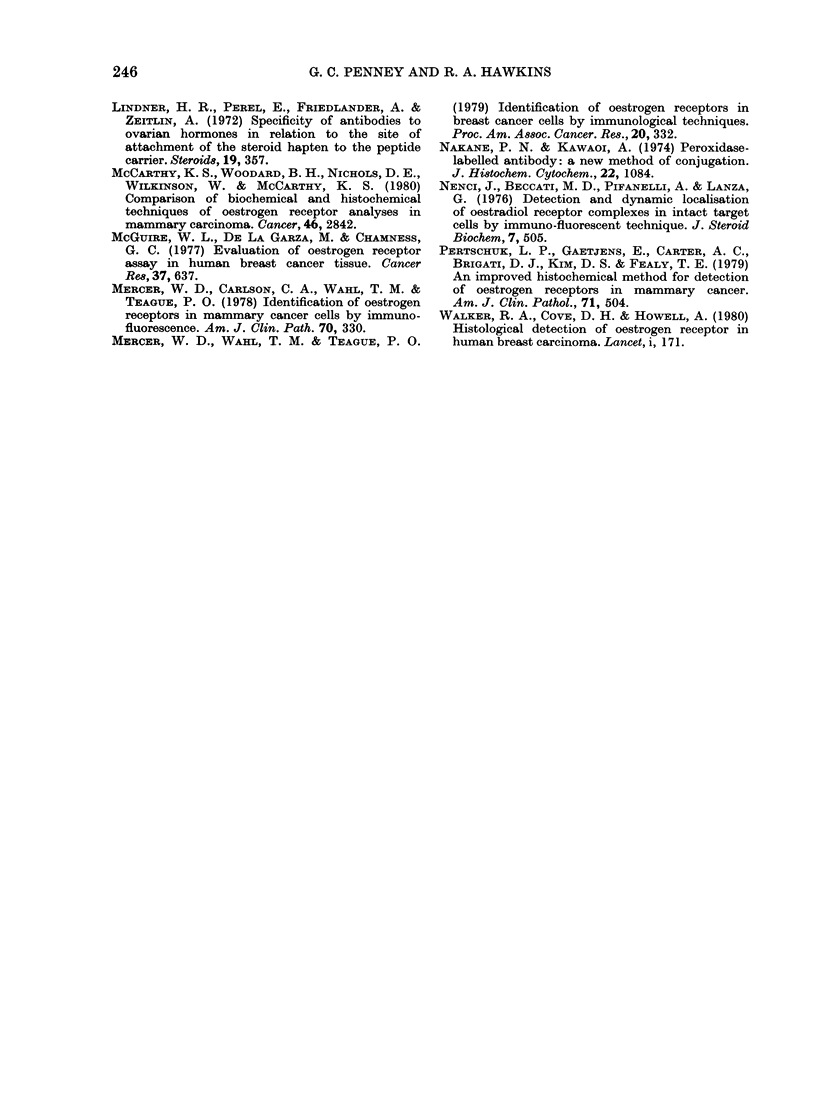# Histochemical detection of oestrogen receptors: a progress report.

**DOI:** 10.1038/bjc.1982.39

**Published:** 1982-02

**Authors:** G. C. Penney, R. A. Hawkins

## Abstract

Four albumin conjugates of oestradiol. Labelled with fluorescein or peroxidase to permit visualization under light or fluorescence microscopy, were synthesized. These were used to examine the feasibility of identifying oestrogen binding in frozen section by two published histochemical techniques. In a variety of experimental tissues and human breast cancers, binding of the oestrogen conjugates was demonstrable, but it appeared nonspecific (i.e., rarely displaceable by competitor) and unrelated to oestrogen receptor (RE) status of the tissue as determined biochemically by assay with dextran-coated charcoal. Investigation of the fate of the RE through the various steps of a histochemical assay, demonstrated major losses of RE from unfixed tissue or after tissue fixation. The RE also exhibited a 10-50-fold poorer affinity for the conjugates synthesized than for oestradiol-17 beta and, at the concentrations of conjugate routinely used in histochemical assays, it seems likely that considerable nonspecific binding takes place. These factors may combine to make it (1) difficult to implement such histochemical assays and (2) unlikely that the RE is being detected.


*Br. J. Cancer* (1982) **45**, 237

### HISTOCHEMICAL DETECTION OF OESTROGEN RECEPTORS: A PROGRESS REPORT

G. C. PENNEY AND R. A. HAWKINS

*From the University Department of Clinical Surgery, Royal Infirmary, Edinburgh*

Received 5 August 1981 Accepted 27 October 1981

**Summary.—Four albumin conjugates of oestradiol. labelled with fluorescein or peroxidase to permit visualization under light or fluorescence microscopy, were synthesized. These were used to examine the feasibility of identifying oestrogen binding in frozen section by two published histochemical techniques. In a variety of experimental tissues and human breast cancers, binding of the oestrogen conjugates was demonstrable, but it appeared nonspecific (*i.e.*, rarely displaceable by competitor) and unrelated to oestrogen receptor (RE) status of the tissue as determined biochemically by assay with dextran-coated charcoal.**

**Investigation of the fate of the RE through the various steps of a histochemical assay, demonstrated major losses of RE from unfixed tissue or after tissue fixation. The RE also exhibited a 10–50-fold poorer affinity for the conjugates synthesized than for oestradiol-17$\beta$ and, at the concentrations of conjugate routinely used in histochemical assays, it seems likely that considerable nonspecific binding takes place. These factors may combine to make it (1) difficult to implement such histochemical assays and (2) unlikely that the RE is being detected.**

It is now well established that the determination of the oestrogen receptor (RE) content of breast-cancer specimens is of value in planning therapy (Hawkins *et al.*, 1980). Techniques in current use for RE assay involve the homogenization of a sizeable portion of tumour (160–250 mg preferred in our laboratory) and are expensive in terms of equipment, radioactive reagents and technicians' time. Recognition of these and other limitations of current biochemical methods has led several groups to investigate techniques for the identification of RE in frozen section by immunohistological or histochemical means (Nenci *et al.*, 1976; Mercer *et al.*, 1978, 1979; Pertschuk *et al.*, 1979; Lee, 1979; Walker *et al.*, 1980). Using histochemical methods, at least two groups (Pertschuk *et al.*, 1979; Walker *et al.*, 1980) have reported a good correlation with the results of biochemical RE assays using dextran-coated charcoal (DCC) or sucrose density gradient (SDG). Such histochemical techniques are of potential clinical value, since little tumour tissue is required and allowance can be made for tumour heterogeneity. In setting up a histochemical system for the identification of the RE protein, several technical and theoretical problems must be overcome Firstly, the fact that RE is a component of the soluble cytoplasm may allow it to "leach out" into aqueous processing media from unfixed cells disrupted by the cutting of frozen sections; this possibility has recently been discussed by McCarthy *et al.* (1980). Secondly, the ability of RE to bind to oestrogen is well recognized as a labile property which might be well impaired if fixative were used to immobilize RE within the tissue section. Thirdly, the conjugation of oestradiol to tracers which can be visualized histochemically might seriously impair the binding of the oestradiol moiety to

RE. Lastly, the concentrations of oestradiol conjugates and of competitive receptor-blockers advocated for histochemical techniques are so high that binding may not be limited to the high-affinity RE, but might also occur at nonspecific binding sites.

In this paper we report on our experience with the histochemical techniques of Pertschuk *et al.* (1979) and Walker *et al.* (1980) and also on our findings in relation to the 4 technical problems outlined above.

### MATERIALS AND METHODS

*Oestradiol-17β-BSA-fluorescein conjugates.*—Conjugates were prepared by the mixed-anhydride method of Erlanger *et al.* (1957). Briefly, oestradiol-17β hemisuccinate (Sigma) was activated by reaction with tri-n-butylamine and isobutylchlorocarbonate in dioxane and then bovine serum albumin (BSA) was added to the reaction mixture. After dialysis, the resulting oestradiol–BSA conjugate was allowed to react with fluorescein isothiocyanate (FITC, Baltimore Biological Laboratories) and again dialysed. Two conjugate preparations of this type were used. The first was a gift from Dr L. Pertschuk and contained 4 mol of oestradiol (linked *via* the C17 position) and 5 mol of fluorescein per mole of BSA. The second was synthesized by us using the same method. Both were used at a final concentration of 50 μg/ml (equivalent to 370 nM with respect to the entire conjugate molecule). In addition, a conjugate comprising only BSA and FITC was synthesized for the assessment of non-specific fluorescein-labelling of tissues due to binding of BSA.

*Oesteradiol-17β-BSA-peroxidase conjugates.*—Conjugates were prepared by the methods of Nakane & Kawaoi (1974) and of Avrameas & Ternynck (1971). In the former, reactive aldehyde groups were created by reaction of horseradish peroxidase (HRP, type IV, Sigma) with sodium periodate. After dialysis, the resulting aldehyde mixture was allowed to react with 17β-oestradiol-6-0-carboxy-methyloxime-BSA (from Steraloids; 33 mol of steroid/mol BSA). The resulting conjugate was stabilized by sodium borohydride treatment and purified by dialysis and final chromatography on a 100 × 1·2cm column of Sephadex G100 to remove low-mol.-wt precursors. In the second method, the reactive aldehyde groups were created by reaction of HRP with glutaraldehyde, the product of activated peroxidase being purified by chromatography on a 60 × 1·2cm column of Sephadex G25. The purified product was allowed to react with 17β-oestradiol-6-0-carboxy-methyloxime-BSA, and the resulting conjugate was stabilized by the addition of lysine, dialysed and purified by chromatography as above. These methods are reported to combine 1–4 mol of HRP with each mole of the BSA-steroid conjugate. The final conjugate concentrations were determined empirically by preliminary straining of sections of rat uterus: determination of the protein concentration (Bradford, 1976) of these solutions indicated a final concentration of ~1·5μM with respect to the entire conjugate molecule. In all, 3 syntheses were undertaken, one by the first method and two by the second.

*Biochemical assay of RE.*—The DCC assay of Hawkins *et al.* (1977) was used for human and rat tissues with one modification: the assay medium comprised Tris buffer (0·25M sucrose, 10mM tris, 1mM ethylene diamine tetra-acetate, pH 8·0 at 25°C) to which had been added glycerol (10% v/v) and monothioglycerol (1% v/v). Results were expressed as fmoles of RE binding sites/mg tissue, derived by Scatchard analysis.

*Histochemical assessment of oestrogen binding using fluorescein tracer.*—The method of Pertschuk *et al.* (1979) was followed. Briefly, frozen 4μm sections were cut, air-dried on to glass slides and incubated, in a humidifier chamber at room temperature, in solutions of oestradiol–BSA–fluorescein conjugate (50 μg/ml). To demonstrate specificity of binding, additional sections were incubated with conjugate plus an excess (100 μg/ml) of an unlabelled competitive receptor-blocker (the anti-oestrogen CI628 (Parke Davies) or diethylstilboestrol (DES). After incubation, the sections were rinsed, fixed for 10 min in ethanol/acetone, extensively washed and mounted in buffered glycerol. The sections were then examined under incident UV light for cellular fluorescence, which was diminished in control sections incubated in the presence of both conjugate and competitor. Further control sections were incubated with a solution of BSA–fluorescein (50 μg/ml).

*Histochemical assessment of oestrogen binding using peroxidase tracer*.—The method of Walker *et al.* (1980) was followed. Briefly $4\mu m$ frozen sections, mounted on glass slides, were fixed by immersion in acetone for 4 min at room temperature. The sections were then incubated for 2 h in a humidifier chamber at room temperature in solutions of BSA–peroxidase conjugate ($\sim 1{\cdot}5$ $\mu$M). Control sections were incubated with conjugate plus an excess of unlabelled, competitive receptor blocker (usually DES, but occasionally CI628 or Tamoxifen) in saturated solution. Following incubation, the slides were extensively washed in PBS and exposed to diaminobenzidine tetrahydrochloride plus $H_2O_2$ for the demonstration of peroxidase. Further controls consisted of similar sections exposed directly to the diaminobenzidine mixture to demonstrate endogenous peroxidase. All sections were again washed, counterstained with haematoxylin, dehydrated, cleared and mounted for light microscopy.

A series of tissues were assayed for RE by the biochemical method and also processed histochemically. The slides were scored for intensity of staining by one of us (G.C.P.) without prior knowledge of the biochemical result.

*Investigation of losses of RE during section cutting*.—To study possible losses of RE due to histochemical processing of specimens, RE-rich tissues (rat uteri and DMBA-induced rat mammary tumours) were assayed for RE with and without being subjected to various steps of histochemical techniques. Firstly, the effect of simply cutting a portion of tissue into $4\mu m$ frozen sections and, secondly, the effect of exposing unfixed $4\mu m$ and $14\mu m$ sections to aqueous buffer were investigated. In the latter case, both the sections and the buffers to which they had been exposed were assayed for RE.

*Investigation of losses of RE during fixation*.—Two different fixation processes were studied with regard to their effects on RE: firstly, fixation in acetone for 4 min before incubating the tissue with steroid (Walker *et al.*, 1980) and secondly, fixation in ethanol/acetone for 10 min *after* incubation (Pertschuk *et al.*, 1979).

Rat uteri were exposed to acetone, homogenized in buffer and centrifuged to yield a cytosol and an insoluble pellet. Both the cytosol and the pellet were then assayed for RE, the first by the standard biochemical method and the latter by incubation of aliquots of the pellet with mixtures of tritiated and unlabelled oestradiol, followed by removal of unbound steroid by washing the pellet in buffer (based on the method of Anderson *et al.*, 1972).

To study fixation with ethanol/acetone, an RE-rich tissue (DMBA-induced rat mammary tumour) was cut into slices ($\sim 0{\cdot}5$ mm thick) which were incubated for 1 h at 25°C in Krebs bicarbonate buffer containing $^3$H-oestradiol (0·18 nM) with or without excess unlabelled oestradiol (92 nM). (These conditions should effect a specific uptake of $^3$H-oestradiol into the cell nucleus—Hawkins *et al.*, 1978). After incubation and brief rinsing in buffer, the slices were immersed in the fixative for 10 min, then extensively washed in buffer. Control slices were processed similarly but omitting the fixation step. All slices were then digested in 5N NaOH and mixed with aqueous scintillator to permit determination of $^3$H uptake by scintillation counting.

*Determination of relative binding affinities of oestradiol conjugates and parent compounds*.—Successive steps in the synthesis of conjugates of oestradiol with tracers suitable for histochemical localization increase the size of the molecule. The effects of these steps on the ability of the 17β-oestradiol moiety to bind to RE were examined by competitive-binding studies. Varying concentrations of the compound under test (0·01 to 10,000 molar excess) were allowed to compete with a fixed concentration of $^3$H-oestradiol (0·03 nM) for binding to aliquots of an RE-rich cytosol, prepared from pooled rat uteri, during an overnight incubation at 4°C. Free and bound hormone were then separated by charcoal adsorption, as described by Hawkins *et al.* (1977).

The increasing displacement of $^3$H-oestradiol from cytosolic receptor sites by increasing concentrations of the various test compounds was plotted and compared with the displacement produced by equivalent concentrations of 17β-oestradiol. Relative binding affinities (RA) were then calculated as:

$$\frac{\text{concentration of } 17\beta\text{-oestradiol for } 50\% \text{ displacement}}{\text{concentration of test compound for } 50\% \text{ displacement}}$$

### RESULTS

### *Histochemical assessment of oestrogen-binding using fluorescein tracer*

The histochemical technique of Pertschuk *et al.* (1979) was applied to a range of human and rat tissues which had been designated $RE^+$ or $RE^-$ by biochemical assay. Morphologically similar $RE^+$ and $RE^-$ tissues (rat uterus and duodenum) exhibited widespread fluorescent labelling of remarkably similar distribution. In neither case was the fluorescence diminished by the presence of either DES or the anti-oestrogen, CI 628, even when the concentration of competitor was increased to the point of sturation. Widespread fluorescent labelling which could not be "blocked" by the presence of competitors was also seen in all speci-

TABLE I.—*Summary of results of DCC assays for RE and of histochemical staining using oestradiol–BSA–peroxidase in* 17 *tissues without endogenous peroxidase.*

| Subject | Tissue | DCC assay result* | Histochemical assay result: Assay no. | Histochemical assay result: Degree of peroxidase staining (0 to + + +) | Blocking by competitor** | Agreement between DCC and histochemical assays |
|---|---|---|---|---|---|---|
| Rat A (lactating) | Uterus | 14·9 | 1 | + + + mainly in endometrium | partial | + |
| | | | 2 | + + + mainly in endometrium | none | ? |
| Rat B (lactating) | Uterus | 13·9 | 1 | + + + all layers | none | ? |
| | | | 2 | + all layers | ?† | ? |
| | | | 3 | + + + all layers | ? | ? |
| Rat C (immature) | Uterus | 13·3 | 1 | + + all layers | none | ? |
| Rat D (immature) | Uterus | 10·2 | 1 | + + + mainly in ectocervical squamous epithelium | partial | + |
| Patient 1 | Ovarian metastatis (L) from breast cancer | 7·4 | 1 | + + + | ? | ? |
| Patient 2 | 1° Breast cancer | 6·0 | 1 | + | none | ? |
| Patient 3 | 1° Breast cancer | 5·0 | 1 | + | ? | ? |
| Patient 4 | 1° Breast cancer | 4·7 | 1 | + + (stroma > cells) | ? | ? |
| Patient 5 | 1° Breast cancer | 3·6 | 1 | + + + within cancer cells only | ? | ? |
| | | | 2 | + + + within cancer cells only | ? | ? |
| | | | 3 | + + + within cancer cells only | total | ? |
| Patient 1 | Ovarian metastasis (R) | 1·7 | 1 | + + | partial | + |
| | | | 2 | 0 | — | — |
| | | | 3 | 0 | — | — |
| | | | 4 | + | ? | ? |
| Patient 6 | 1° Breast cancer | 1·7 | 1 | ± (stroma > cells) | ? | ? |
| Patient 7 | Benign mammary dysplasia | 0·6 | 1 | 0 | — | + |
| Patient 8 | 1° Breast cancer | 0 | 1 | 0 | — | + |
| Patient 9 | 1° Breast cancer | 0 | 1 | + + | none | — |
| Patient 10 | Cytosarcoma | 0 | 1 | + + + | none | — |
| Rat E | Squamous epithelium of ear | 0 | 1 | + + + | none | — |
| Rat F | Cheek muscle | 0 | 1 | + + | none | — |

* fmoles receptor/mg tissue.

** The competitor used was a saturated solution of DES in 21 assays, or Tamoxifen in 2 assays and of CI-628 in 2 assays.

† Indicates that tissue architecture was disrupted by high concentration of competitor, making assessment of blocking impossible.

‡ Agreement between the two assay techniques has been assessed as follows:

+ Positive staining with convincing blocking in tissues designated RE-rich by DCC assay or absent staining in tissues designated RE-poor by DCC assay.

— Absent staining in tissues designated RE-rich by DCC assay or positive staining in tissues designated RE-poor by DCC assay.

? Positive staining but unconvincing blocking in tissues designated RE-rich on DCC assay.

mens of human and rat mammary tumours examined. Incubation of sections with oestrogen-free, BSA–fluorescein conjugate also produced widespread, but slightly less intense, fluorescence.

*Histochemical assessment of oestrogen-binding using peroxidase tracer*

All 3 synthesized preparations of oestradiol–BSA–peroxidase conjugate were effective in producing some staining of sections at dilutions of 1/8 to 1/20 of the final eluate from gel filtration. Higher concentrations resulted in heavy deposition over the entire section. Various human and rat tissues were studied by the method of Walker *et al.* (1980). Staining due to endogenous peroxidase was found to be abundant in sections of human uterus and rat duodenum, and these tissues were therefore considered unsuitable for study by the peroxidase tracer technique, as it would have proved impossible to distinguish between "endogenous" and "tracer" peroxidases. The microscopic appearances found in 25 histochemical assays on 17 tissues, in which interpretation was not complicated by the presence of endogenous peroxidase staining, are summarized in Table I. In all 19 assays on tissues which had high or moderate RE levels on biochemical assessment, cellular uptake of conjugates was demonstrable. However, reproducibility was poor, and in only 4 of those 19 cases could such uptake be diminished by the presence of an unlabelled competitor ("blocking"). Even in these 4 cases, the competitor had to be used in saturated solution to achieve blocking. Cellular uptake of conjugate was also demonstrable in many tissues which were RE poor by biochemical assay; blocking was not demonstrable in any of these tissues. Cellular uptake of conjugate was absent from only 2 of the 6 tissues which were RE-poor biochemically. Overall, therefore, agreement with biochemical assay was obtained in only 6/25 assays: 4 RE-rich tissues where cellular uptake occurred, and could be blocked, and 2 RE-poor tissues where no uptake occurred.

*Loss of RE from unfixed tissues*

Portions of rat uterus were homogenized and assayed for RE, "intact" or after cutting into 4μm cryostat sections. This revealed a concentration of RE in the "intact" portion of 6·69 fmol/mg tissue, but only 2·45 fmol/mg tissue in the "sectioned" portion, a loss of over 50% of detectable RE.

Frozen sections of tissue were exposed to aqueous processing media and RE assays performed on the sections and media after separation by gentle centrifugation. The results of these assays, on 4μm and 14μm sections, and their respective media, are shown in Fig 1. In the case of 4μm sections, RE activity in the washing buffer was 4× that remaining in the sections and, in the case of 14μm sections, was 3× as great. Thus, it is evident that RE is very readily lost from unfixed tissues.

FIG. 1.—Scatchard plots of data from RE assays performed on cytosols prepared from 4μm and 14μm frozen sections of DMBA-induced rat mammary tumour and on aliquots of buffer in which the sections had been washed. ○, washing buffer from 4μm sections. △, washing buffer from 14μm sections. ▲, cytosol prepared from 14μm sections. ●, cytosol prepared from 4μm sections.

FIG. 2.—Scatchard plots of data from RE assays performed on cytosols and pellets prepared from rat uteri, after fixation in acetone or unfixed. ○, cytosol prepared from unfixed uteri. ●, pellet prepared from acetone-fixed uteri. △, cytosol prepared from acetone-fixed uteri. (No RE was detectable in the pellet prepared from unfixed uteri). N.B. Results of cytosol and pellet assays are not directly comparable as reagent concentrations were not identical.

*Loss of RE during fixation*

*Acetone.*—When rat uteri were exposed to either acetone or buffer for 4 min, homogenized and separated into cytosol and pellet for RE-assay, no activity was detectable in the pellet preparation from the unfixed (buffer-exposed) specimen. The other 3 preparations (acetone-fixed cytosol, acetone-fixed pellet and unfixed cytosol) contained RE and Scatchard plots for the assays of these are reproduced in Fig. 2. After acetone fixation, both concentration and apparent affinity of RE binding were markedly impaired, by comparison with the cytosolic RE of the unfixed specimen. The total RE concentration (pellet plus cytosol) detectable in the acetone-treated specimen was less than half that detectable in the cytosol from the untreated tissue. Thus, acetone-fixation appears to destroy at least 50% of the receptor present.

*Ethanol/Acetone.*—In unfixed 0·5mm tissue slices of DMBA-induced tumour, marked uptake of $^3$H occurred after incubation with $^3$H-oestradiol alone. Such uptake was diminished by almost 75% in slices incubated with $^3$H-oestradiol plus excess, unlabelled oestradiol. This differential labelling was regarded as indicative of RE. In tissue slices which had been fixed in ethanol/acetone after incubation with steroid, minimal uptake of $^3$H-oestradiol was detectable, and there was no differential labelling between slices incubated with $^3$H-oestradiol alone and those incubated in the presence of unlabelled oestradiol (see Table II). This finding suggested that any binding of steroid to tissue which had occurred during the incubation period had been abolished by exposure to the fixative.

TABLE II.—*The effect of ethanol/acetone post-fixation on uptake of oestradiol 17β by slices of DMBA-induced rat mammary tumour. Each result represents the mean of 2 similarly treated flasks of sections*

| Form of fixation | Steroid content of incubation medium | Counts bound (ct/min/mg tissue) |
|---|---|---|
| None | $^3$H-oestradiol only | 183 |
| | labelled and excess unlabelled oestradiol | 51 |
| Ethanol/acetone | $^3$H-oestradiol only | 4 |
| | labelled and excess unlabelled oestradiol | 6 |

*Relative binding affinities (RA) of oestradiol conjugates and parent compounds*

The following 7 compounds were subjected to competitive-binding studies: 17β-oestradiol, the parent molecule; two prepara-

TABLE III.—*Relative binding affinities (RA) of conjugates and conjugate precursors used for RE histochemistry. Binding affinities relate to the molar concentration of each compound which causes 50% inhibition of $^3$H-oestradiol binding, on a basis of oestradiol 17β = 1 (where conjugate concentrations refer to molarity of the entire conjugate molecule rather than oestradiol moieties).*

| Compound | RA |
|---|---|
| Oestradiol 17β | 1·0 |
| Oestradiol–BSA–Fluorescein (Pertschuk) | 0·8 |
| Oestradiol–BSA–Fluorescein (present authors) | 0 05 |
| BSA-Fluorescein | 0·00 |
| 6-keto-oestradiol 17β | 0·13 |
| 6-keto-oestradiol–BSA | 0·09 |
| 6-keto-oestradiol–BSA–peroxidase | 0·02 |

FIG. 3.—Curves illustrating the binding of $C_{17}$-linked oestradiol conjugates and of a conjugate of BSA and fluorescein alone to the RE, relative to that for oestradiol 17$\beta$. Curves show the increasing displacement of a fixed concentration (0·03nM) of $^3$H-oestradiol by increasing concentrations of the compound under test. Figures on the vertical axis indicate the binding of $^3$H-oestradiol as a percentage of the total bound in the absence of competitor. ○——○, Oestradiol-17$\beta$. □——□, Oestradiol–BSA–Fluorescein—supplied by L. Pertschuk. △——△, Oestradiol–BSA–Fluorescein—synthesized in our lab. ▲——▲, BSA–Fluorescein—synthesized in our lab.

FIG. 4.—Curves illustrating the binding of $C_6$-linked oestradiol conjugates to the RE, relative to that for oestradiol 17$\beta$. Curves show the increasing displacement of a fixed concentration (0·03nM) of $^3$H-oestradiol by increasing concentrations of the compound under test. Figures on the vertical axis indicate the binding of $^3$H-oestradiol as a percentage of the total bound in the absence of competitor. ○, oestradiol 17$\beta$. ×, 6-keto-oestradiol 17$\beta$. △, 6-keto-oestradiol–BSA. □, 6-keto-oestradiol–BSA–peroxidase.

tions of oestradiol–BSA–fluorescein conjugated *via* $C_{17}$ (one synthesized by us and one donated by Dr L. Pertschuk); a conjugate of BSA and fluorescein alone; 6-keto-oestradiol, the precursor of conjugates utilizing the $C_6$ position; oestradiol–BSA conjugated *via* $C_6$ (Steraloids) and oestradiol–BSA–peroxidase (synthesized by us from this precursor). Binding curves for the $C_{17}$ and $C_6$ conjugates are shown in Figs 3 & 4 respectively, and the RAs of the compounds tested are listed in Table III. Successive conjugations to the 17$\beta$-oestradiol moiety via $C_6$ diminished the RA. The conjugates prepared *via* $C_{17}$, particularly that donated by Dr L. Pertschuk, exhibited higher binding affinities.

### DISCUSSION

### *Histochemical technique in practice*

In our hands, the main problems encountered during attempts to reproduce the fluorescein tracer technique of Pertschuk *et al.* (1979) was the widespread uptake of conjugate, which could not be diminished by the presence of competitors, in all the tissues studied. This finding may be related to the form of fixation used in this technique. The experiment described in this paper, in which $^3$H-oestradiol was used to study the effects of ethanol/acetone fixation on RE binding, suggests that such fixation might cause disruption of the oestradiol–RE bond. Thus, fixation in ethanol/acetone after incubation of frozen sections with conjugate (as in Pertschuk's technique) might disrupt any binding to RE which has occurred during the incubation period, as well as serving to immobilize the protein-containing conjugate wherever it lies in the section.

Attempts to reproduce the peroxidase tracer technique of Walker *et al.* (1980) resulted in binding of conjugate which did, in some instances, appear to be "specific", in that it was diminished by the presence of a competitor. However, when the method was repeated on the same tissue on several occasions, reproducibility was poor, as was correlation between the results of the histochemical technique and those of biochemical assays. These disappointing results may have arisen from a combination of the losses of RE accruing from histochemical proces-

TABLE IV.—*Summary of the sites and magnitudes of losses of RE which may occur when tissues are exposed to histochemical processing.*

| Process | Author | % Loss |
|---|---|---|
| Section cutting | Present work | 50 |
| Exposure of unfixed tissue to aqueous media | Present work | 66–75 |
| Fixation in acetone | Present work | 50 |
| Fixation in acetone | Lee (1978) | "Complete" |
| Fixation in glutaraldehyde | Lee (1978) | "Complete" |
| Fixation in formaldehyde | Lee (1978) | "Complete" |
| Fixation in formaldehyde | Dandliker *et al.* (1978) | 60–80 |
| "Post-fixation" in ethanol/acetone | Present work | Almost total |

sing, the low binding affinities of oestradiol conjugates and a high degree of non-specific binding resulting from the use of high conjugate concentrations.

*Losses of RE*

Losses of RE may occur at several of the stages of histochemical procedures. These losses, as calculated from the present work and from the studies of others, are summarized in Table IV. In our hands, the steps involved in the technique of Walker *et al.* (1980) might allow some 25% of the RE originally in the tissue to remain "viable", but a procedure incorporating the ethanol/acetone fixation step of Pertschuk *et al.* (1979) might totally abolish any specific RE activity.

*Binding affinities of conjugates*

Conjugates which have been advocated for RE histochemistry can be divided into two main groups: those where the oestradiol moiety is linked to the tracer *via* the $C_6$ position (*e.g.* that of Walker *et al.*, 1980) and those linked *via* $C_{17}$ (*e.g.* that of Pertschuk *et al.*, 1979). Theoretical considerations concerning the postulated site of the oestradiol–RE bond (Ellis & Ringold, 1971) and the maintenance of the antigenecity of oestradiol (Lindner *et al.*, 1972) would favour the $C_6$ position as the site of conjugation. However our own results (Table III) and those of Dandliker *et al.* (1978) indicate that, in practice, conjugation *via* $C_{17}$ shows superior binding affinities. Indeed, the RA of 0·8 obtained for the sample of oestradiol–BSA–fluorescein provided by Dr L. Pertschuk is strikingly superior to those of the other compounds examined. However, this RA must be interpreted with some caution, in view of the point raised by Dandliker *et al.* (1978) that a small amount of an active contaminant, or a small amount of degradation liberating free, unlabelled hormone, could lead to an inflated estimate of RA.

The RA of only 0·02 obtained for the $C_6$-linked conjugate of Walker *et al.* may account, at least in part, for the disappointing results with their technique It was encouraging to note that the compound containing only BSA and fluorescein failed to inhibit binding of $^3H$-oestradiol to RE, which suggests that the inhibition produced by the oestradiol-containing conjugates was not non-specific, *i.e.* due simply to the presence of material of high molecular weight.

*Concentration of conjugates*

For RE assay systems in general, it is felt that a concentration of labelled oestradiol which is adequate to saturate the receptors, but not vastly in excess of the saturating concentration, should be used. Concentrations of 1–5nM (McGuire *et al.*, 1977; King *et al.*, 1979) have been advocated as appropriate for biochemical techniques using a single, saturating dose of labelled oestradiol. If a concentration vastly in excess of that needed for saturation is used, binding to non-specific proteins (*e.g.* albumin, Kd=

$10^{-4}$ to $10^{-5}$) is increased. Such binding may be confused with specific RE, in that it may be inhibited by the presence of an even higher concentration of competitor.

The concentrations of labelled oestradiol in the various histochemical techniques are very much higher than the 1–5nM suggested for RE saturation. Pertschuk *et al.* (1979) used a conjugate concentration of 370 nM (1480 nM with respect to the oestradiol moieties). Lee (1979) used a conjugate concentration of 46 $\mu$M (1·1 mM with respect to oestradiol). The approximate conjugate and oestradiol concentrations used by Walker *et al.* (1980) were 1·5 $\mu$M and 50 $\mu$M respectively (estimates based on protein assays carried out by the present authors). At concentrations such as these, binding to low-affinity sites would almost certainly occur, leading to difficulties in interpreting the results.

In summary, there is wide agreement that a reliable and reproducible technique for the identification of RE in histological sections would represent a significant advance over currently available biochemical assays. From the good correlation with the results of biochemical assays reported by at least two groups, it would seem that such a histochemical technique may, indeed, be a reality. However, the losses of RE inherent in histochemical processing, the low binding affinities of many oestradiol conjugates, and the inconsistency between the concentrations of conjugates and competitors used and the known binding affinity of RE, must raise serious doubts that true RE is being demonstarated. It seem possible that binding to another oestrogen—binding protein of lower affinity (perhaps the Type II or Type III receptor suggested by Chamness *et al.*, 1980) is being demonstrated, and that binding to this protein may, in turn, correlate with biochemically—estimated RE activity. However, the difficulties which we have experienced, plus the lack of reported success with such methods from other centres, would argue that, at the present time, histochemical detection of oestrogen—binding is technically too difficult for general use.

We would like to thank Professor A. P. M. Forrest for his support and encouragement, Mr Walter Hawkins for technical assistance in frozen section cutting, Dr L. P. Pertschuk, Department of Pathology, King's County Hospital, Brooklyn, New York, for a gift of his conjugate, Dr R. A. Walker, Department of Pathology, University of Birmingham, for personal instruction in her method, Professor C. W. Horn, Department of Pathology, University of Aberdeen, for helpful discussions and the Tenovus organization for grant support to G.C.P.

### REFERENCES

Anderson, J., Clark, J. H. & Peck, E. J. (1972) Oestrogen and nuclear binding sites: Determination for specific sites by ($^3$H) oestradiol exchange. *Biochem. J.*, **126**, 561.

Avrameas, S., & Ternynck, T. (1971) Peroxidase labelled antibody and Fab conjugates with enhanced intracellular penetration. *Immunology*, **8**, 1175.

Bradford, M. M. (1976) A rapid and sensitive method for the quantitation of microgram quantities of protein utilizing the principle of protein–dye binding. *Anal Biochem*, **72**, 248.

Chamness, G. C., Mercer, W. D. & McGuire, W. L. (1980) Are histochemical methods for oestrogen receptor valid. *J. Histochem. Cytochem.*, **28**, 792.

Dandliker, W. B., Brawn, R. J., Hsu, M. L., Levin, J., Meyers, C. Y. & Kolb, V. M. (1978) Investigation of hormone-receptor interactions by means of fluorescence labelling. *Cancer Res.*, **38**, 4212.

Ellis, D. J. & Ringold, H. J. (1971) The uterine oestrogen receptor: A physicochemical study. In *The Sex Steroids* (Ed. McKerns). New York: Appleton-Century-Crofts. p. 73.

Erlanger, B. F., Borek, E., Beister, S. M. & Lieberman, S. (1957) Preparation and characterisation of conjugates of BSA with testosterone and cortisone. *J. Biol. Chem.*, **228**, 713.

Hawkins, R. A., Hill, A., Freedman, B. & 4 others (1977) Oestrogen receptor activity and endocrine status in DMBA-induced rat mammary tumours. *Eur. J. Cancer*, **13**, 223.

Hawkins, R. A., Roberts, M. M. & Forrest, A. P. M. (1980) Oestrogen receptors and breast cancer: Current status. *Br. J. Surg*, **67**, 153.

Hawkins, R. A., Scott, K. M. & Freedman, B. (1978) Oestrogen receptor activity and intramuscular translocation in ovarian-dependent and independant rat mammary tumours. *J. Endocrinol.*, **77**, 63P.

King, R. J. B., Redgrave, S., Hayward, J. L., Millis, R. R. & Rubens, R. D. (1979) The measurement of receptors for oestrogen and progesterone in human breast tumours. In *Steroid Receptor Assays in Human Breast Tumours: Methodological and Clinical Aspects* (Ed. King). Cardiff: Alpha Omega.

Lee, S. H. (1978) Cytochemical study of oestrogen receptor in human mammary cancer. *Am. J. Clin. Pathol.*, **70**, 197.

LINDNER, H. R., PEREL, E., FRIEDLANDER, A. & ZEITLIN, A. (1972) Specificity of antibodies to ovarian hormones in relation to the site of attachment of the steroid hapten to the peptide carrier. *Steroids*, **19**, 357.

MCCARTHY, K. S., WOODARD, B. H., NICHOLS, D. E., WILKINSON, W. & MCCARTHY, K. S. (1980) Comparison of biochemical and histochemical techniques of oestrogen receptor analyses in mammary carcinoma. *Cancer*, **46**, 2842.

MCGUIRE, W. L., DE LA GARZA, M. & CHAMNESS, G. C. (1977) Evaluation of oestrogen receptor assay in human breast cancer tissue. *Cancer Res*, **37**, 637.

MERCER, W. D., CARLSON, C. A., WAHL, T. M. & TEAGUE, P. O. (1978) Identification of oestrogen receptors in mammary cancer cells by immunofluorescence. *Am. J. Clin. Path.* **70**, 330.

MERCER, W. D., WAHL, T. M. & TEAGUE, P. O. (1979) Identification of oestrogen receptors in breast cancer cells by immunological techniques. *Proc. Am. Assoc. Cancer. Res.*, **20**, 332.

NAKANE, P. N. & KAWAOI, A. (1974) Peroxidase-labelled antibody: a new method of conjugation. *J. Histochem. Cytochem.*, **22**, 1084.

NENCI, J., BECCATI, M. D., PIFANELLI, A. & LANZA, G. (1976) Detection and dynamic localisation of oestradiol receptor complexes in intact target cells by immuno-fluorescent technique. *J. Steroid Biochem*, **7**, 505.

PERTSCHUK, L. P., GAETJENS, E., CARTER, A. C., BRIGATI, D. J., KIM, D. S. & FEALY, T. E. (1979) An improved histochemical method for detection of oestrogen receptors in mammary cancer. *Am. J. Clin. Pathol.*, **71**, 504.

WALKER, R. A., COVE, D. H. & HOWELL, A. (1980) Histological detection of oestrogen receptor in human breast carcinoma. *Lancet*, i, 171.